# Exploring the role of immune pathways in the risk and development of depression in adolescence: Research protocol of the IDEA-FLAME study

**DOI:** 10.1016/j.bbih.2021.100396

**Published:** 2021-11-27

**Authors:** Valeria Mondelli, Annamaria Cattaneo, Naghmeh Nikkheslat, Laila Souza, Annabel Walsh, Zuzanna Zajkowska, Valentina Zonca, Moira Marizzoni, Helen L. Fisher, Brandon A. Kohrt, Christian Kieling, Paola Di Meglio

**Affiliations:** a King's College London, Department of Psychological Medicine, Institute of Psychiatry, Psychology, London, UK; b National Institute for Health Research Mental Health Biomedical Research Centre, South London and Maudsley NHS Foundation Trust and King's College London, London, UK; c Department of Pharmacological and Biomolecular Sciences, University of Milan, Via Balzaretti 9, 20133, Milan, Italy; d Laboratory of Biological Psychiatry, IRCCS Istituto Centro San Giovanni di Dio Fatebenefratelli, Brescia, Italy; e Departamento de Psiquiatria, Universidade Federal do Rio Grande do Sul (UFRGS), Porto Alegre, Brazil; f King's College London, Social, Genetic & Developmental Psychiatry Centre, Institute of Psychiatry, Psychology & Neuroscience, London, UK; g ESRC Centre for Society and Mental Health, King's College London, London, UK; h Division of Global Mental Health, Department of Psychiatry, School of Medicine and Health Sciences, The George Washington University, Washington, DC, USA; i Serviço de Psiquiatria da Infância e Adolescência, Hospital de Clínicas de Porto Alegre (HCPA), Porto Alegre, Brazil; j St John's Institute of Dermatology, King's College London, London, UK; k National Institute for Health Research Mental Health Biomedical Research Centre, Guy's and St Thomas' NHS Foundation Trust and King's College London, London, UK

**Keywords:** Depression, Adolescence, Cytokines, RNAseq, Flow cytometry, Immune cells

## Abstract

Extensive research suggests a role for the innate immune system in the pathogenesis of depression, but most of the studies are conducted in adult populations, in high-income countries and mainly focus on the study of inflammatory proteins alone, which provides only a limited understanding of the immune pathways involved in the development of depression. The IDEA-FLAME study aims to identify immune phenotypes underlying increased risk of developing depression in adolescence in a middle-income country. To this end, we will perform deep-immunophenotyping of peripheral blood mononuclear cells and RNA genome-wide gene expression analyses in a longitudinal cohort of Brazilian adolescents stratified for depression risk. The project will involve the 3-year follow-up of an already recruited cohort of 150 Brazilian adolescents selected for risk/presence of depression on the basis of a composite risk score we developed using sociodemographic characteristics (50 adolescents with low-risk and 50 with high-risk of developing depression, and 50 adolescents with a current major depressive disorder). We will 1) test whether the risk group classification at baseline is associated with differences in immune cell frequency, phenotype and functional status, 2) test whether baseline immune markers (cytokines and immune cell markers) are associated with severity of depression at 3-year follow-up, and 3) identify changes in gene expression of immune pathways over the 3-year follow-up in adolescents with increased risk and presence of depression. Because of the exploratory nature of the study, the findings would need to be replicated in a separate and larger sample. Ultimately, this research will contribute to elucidating key immune therapeutic targets and inform the development of interventions to prevent onset of depression among adolescents.

## Highlights

• IDEA-FLAME study aims to identify immune phenotypes of depression risk in adolescence.
• IDEA-RiSCo is a cohort of adolescents stratified for risk of developing depression.
• The study will help to identify immune therapeutic targets for adolescent depression.

## 1 Introduction

Depression is a leading cause of disability in adolescents worldwide and is a major risk factor for suicide, the third most frequent cause of death in adolescents globally (Thapar et al., 2012; Ferrari et al., 2013). For adolescents suffering with depression the benefits of currently available treatments are modest at best (Cipriani et al., 2016) and large randomised controlled trials have failed to demonstrate the efficacy of universal strategies for the prevention of depressive disorder (Stallard et al., 2012). Widespread classroom-based prevention programmes directed at adolescents, regardless of their risk status, have shown more modest effects than programmes designed to target individuals at higher risk of developing depression (Stallard et al., 2012). A barrier to developing effective preventative interventions for adolescent depression is that we lack a sufficient understanding of how depression develops during adolescence and how to identify adolescents who are at highest risk for depression to enable targeted prevention.

Extensive research, mainly conducted in adult populations from high-income countries (HICs), suggests a role for inflammation and the innate immune system in the pathogenesis of depression (Miller and Raison, 2016). Adult patients with major depression show increased levels of inflammatory cytokines including interleukin (IL)-6 and tumor necrosis factor (TNF) in peripheral blood and cerebrospinal fluid (Baumeister et al., 2014; Enache et al., 2019). However, studies in adults cannot inform us about the inflammatory mechanisms involved in the early stages of the disorder and more specifically during adolescence when most patients experience their first episode of depression (Avenevoli et al., 2015). Despite adolescence being frequently the time of first episode of depression, there is an evident paucity of adolescent mechanistic studies; this can be attributed to multiple factors, including ethical considerations for potential invasive investigations (e.g. blood samples etc.) and difficulties in acquiring consent. These barriers unfortunately contribute to make this area of research less popular and more stigmatized, leading to less funding available for these studies and to widening the gap between adolescent and adult research. So far, only a few studies have investigated immune markers in adolescents with depression, mainly focusing on levels of inflammatory proteins and suggesting a possible increase in TNF (D'Acunto et al., 2019). However, the study of inflammatory proteins alone provides only a limited understanding of the immune pathways involved in the development of depression. Therefore, more research is needed on cellular immune markers and gene expression of immune pathways.

The field of immunopsychiatry is rapidly evolving and holds important potential for developing easy-to-access, mechanistic biomarkers for prevention and treatment of mental health disorders (Caldwell et al., 2020). We have recently shown the potential utility of cellular immune markers to distinguish inflamed and uninflamed subgroups of depression in adult individuals (Lynall et al., 2019). Deep cellular immunophenotyping is an important approach to identifying fundamental causal mechanisms, that could not be implied by limiting our focus on downstream markers, such as cytokines and C-reactive protein. Although activation of innate immune response has largely dominated the landscape of research in depression, some studies have also demonstrated an impairment in acquired immune response involving the potential neuroprotective effects mediated by Treg cells (Miller and Raison, 2016). Our recent findings show an increased frequency of CD4^+^ T cells in adults with depression (Lynall et al., 2019). Therefore, the investigation of phenotypic and functional features of both myeloid and lymphoid immune cells, that we would address with the IDEA-FLAME study, hold the potential to better identify and refine the immune system's role in the pathogenesis of depression in adolescents. On the other hand, transcriptomic analyses, including our own work, has shown abnormal expression of gene networks important for the regulation and implementation of innate immune response in adults with depression (Cattaneo et al., 2018; Wittenberg et al., 2020). In line with recent widely endorsed views encouraging maximising the potential of immunopsychiatry (Caldwell et al., 2020), the IDEA-FLAME research protocol includes application of high-throughput cutting-edge laboratory techniques such as multiparameter flow-cytometry and RNA-Seq to a well-characterised cohort of adolescents stratified by risk of depression.

A stratified medicine approach is needed to transform mental health research and to support more tailored interventions and better targeting of treatments (Schumann et al., 2014). As part of our previous work within the Identifying Depression Early in Adolescence (IDEA) project (Kieling et al., 2019), we generated a composite risk score for the development of depression in adolescence using sociodemographic variables (Botter-Maio Rocha et al., 2020; Brathwaite et al., 2020a). In order to identify specific immune mechanisms associated with increased risk or presence of depression, we will take advantage of the unique cohort (the IDEA - Risk Stratified Cohort (IDEA-RiSCo)) of Brazilian adolescents that has been stratified for risk and presence of depression by using this risk score (Kieling et al., 2021). One of the innovative aspects of this cohort is that it does not assume that adolescents without depression constitute a homogenous group and therefore uses a risk score to stratify for risk of future depression (low vs. high). Most of the currently available mental health samples typically compare cases and non-cases where the latter are defined by lack of a current psychiatric disorder. However, non-cases may have a number of risk factors that make them likely to develop a disorder in the future, leading to a high amount of noise and heterogeneity in these typical designs. This can be rectified by risk stratification of non-cases (into low and high risk), such as has been done in IDEA-RiSCo. Of note, the IDEA risk score includes sociodemographic variables (such as childhood maltreatment, social isolation, drug use etc.) that have previously been associated with increased levels of inflammatory markers (Baumeister et al., 2016; Zilioli and Jiang, 2021; Carbia et al., 2021). It is therefore possible to hypothesise that the high risk, as indicated by sociodemographic variables/IDEA risk score, would contribute to the onset of depression partially through the modification of the immunophenotype, as also suggested in our recently published systematic review (Zajkowska et al., 2021).

The IDEA-FLAME study will focus on this depression risk-stratified cohort of adolescents recruited in Brazil. Ninety percent of the world's adolescents live in low- and middle-income countries (LMICs), yet the majority of research on inflammation and depression has been conducted in HICs (D'Acunto et al., 2019). This highlights a significant gap in our knowledge of immune mechanisms that could help explain how depression develops in adolescents in LMICs, where the psychosocial context often differs from HICs. Our research protocol focusing on the Brazilian IDEA-RiSCo cohort will allow us to fill this gap in existing knowledge (Kieling et al., 2019). Psychosocial context can interact with the immune system to influence the development of depression (Baumeister et al., 2016; Zajkowska et al., 2021); e.g. frequency of circulating monocytes is increased following early experience of social adversities (Mondelli and Vernon, 2019) and increased low-grade systemic inflammation predicts subsequent development of depression only in adolescents with experience of childhood adversities (Miller and Cole, 2012). This highlights the need to identify cross-culturally and cross-contextually immune processes that would increase our understanding of the biological mechanisms by which social experiences impact depression risk.

The purpose of this manuscript is to present the protocol of the IDEA-FLAME study, including details on the scientific methodology, laboratory and statistical analyses to enhance transparency of research, reduce publication bias and prevent selective publication and selective reporting of research outcomes, as well as to support reproducibility of results for future research studies.

## 2 Aims and hypotheses

Our IDEA-FLAME study aims to address some of the above challenges by investigating immune mechanisms underlying increased risk of developing depression in adolescence, by performing deep-immunophenotyping and RNAseq analyses in a longitudinal cohort of adolescents stratified for depression risk. The follow-up of the IDEA-RiSCo cohort has been planned for 3 years after the baseline assessments were conducted because 3 years was the prediction interval for which the IDEA risk score was originally developed (i.e. the score calculated at age 15 predicted increased risk of depression at age 18) (Botter-Maio Rocha et al., 2020). More specifically this study aims to: 1) Test whether increased risk or presence of adolescent depression is associated with differences in immune cell frequency, phenotype and functional status (cross-sectional analyses on baseline samples). 2) Test whether baseline immune markers (cytokines and immune cell markers) are associated with depressive symptoms at follow-up (longitudinal analyses between baseline and 3-year follow-up). 3) Identify changes in immune pathways (or to identify novel pathways) over a 3-year period in adolescents with increased risk and presence of depression vs those with low-risk of depression (longitudinal analyses between baseline and 3-year follow-up).

We hypothesise that: 1) Adolescents with depression and those with high-risk of future depression will present increases in percentage and activity of classical and non-classical monocytes compared with low-risk adolescents. We also hypothesise that depressed adolescents will present with increases in percentage of CD4^+^ T cell when compared with adolescents with low- and high-risk of depression. For the first aim we also plan to take a hypothesis-free approach as we will additionally investigate other cell types and their frequency and phenotype (e.g. dendritic cells activation markers, cytokines production etc). 2) Higher levels of IL-6 and TNF at baseline will be associated with more severe psychopathology at 3-year follow-up. 3) Adolescents with depression and those with high-risk of future depression will present similar changes between baseline and 3-year follow-up in the expression of pathways and networks regulating the innate immune response that will not be apparent in adolescents with low-risk for future depression.

## 3 Methods

IDEA-FLAME is a nested explorative research project within the larger Identifying Depression Early in Adolescence - Risk Stratified Cohort (IDEA-RiSCo) study, which aims to understand the neurobiological mechanisms of adolescent depression risk by studying a longitudinal cohort of 150 adolescents stratified at baseline for risk/presence of depression (50 adolescents with low-risk, 50 adolescents with high-risk of future depression, 50 adolescents with current, untreated, depression) recruited from schools in Porto Alegre in Brazil (Kieling et al., 2021).

### 3.1 Description of IDEA - Risk Stratified Cohort (IDEA-RiSCo)

In order to stratify risk of developing major depression among adolescents, we have previously developed a composite score using data from the Pelotas 1993 Birth Cohort (Goncalves et al., 2014). Using only sociodemographic variables obtained from the adolescent at age 15, we developed the IDEA risk-score, which has good discriminative performance (c-statistic of 0.78) to identify individuals at high-risk for developing major depression at age 18 (Botter-Maio Rocha et al., 2020). In the Pelotas 1993 Birth Cohort, adolescents scoring above the 90^th^ percentile at age 15 years had a 38% probability of depression three years later; among those below the 20^th^ percentile none had a depressive episode at age 18 years. The composite risk score has subsequently been shown to perform beyond chance in other countries (including the UK) (Botter-Maio Rocha et al., 2020; Brathwaite et al., 2020a; Brathwaite et al., 2020b).

**Fig. 1:**
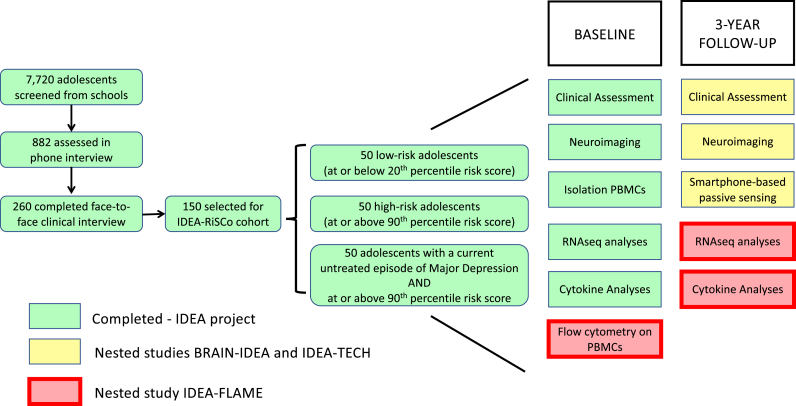
Overview of the IDEA-RiSCo study: recruitment process, the baseline data already available, data collected and analysed as part of other two nested studies (BRAIN-IDEA and IDEA-TECH) and analyses to be conducted as part of IDEA-FLAME.

As part of the IDEA project, we have established a new cohort (IDEA-RiSCo) of 150 Brazilian adolescents that has been stratified for risk and presence of depression by using the IDEA risk-score (see Fig. 1). A total of 7720 adolescents aged 14–16 years of age were initially screened in public state schools in Porto Alegre, Brazil. One hundred and fifty adolescents were selected for inclusion in the study based on their risk scores derived from the screening information and subsequent clinical assessment to check for current and lifetime history of depressive disorders (including dysthymia) using the Brazilian Portuguese translation of the Schedule for Affective Disorders and Schizophrenia for School-Age Children-Present and Lifetime Version (K-SADS-PL) (Caye et al., 2017). At baseline, the 150 adolescents were aged 14–16 years, 50% were female, and 44% of African descent. Participants were classified into one of three groups at baseline: a group of 50 low-risk participants with no current or past depression who were at or below the 20^th^ percentile on the risk score; a group of 50 high-risk participants with no current or past depression at baseline but at or above the 90^th^ percentile on the risk score; and a group of 50 participants with a current, untreated, episode of major depression and at or above the 90^th^ percentile on the risk score (Kieling et al., 2021). To allow for two-by-two comparisons between groups, adolescents with current depression were also required to score at or above the 90^th^ percentile of the risk score.

### 3.2 Clinical assessments and data collection supported by the larger IDEA project

The baseline assessment included a clinical diagnostic interview (K-SADS-PL) (Caye et al., 2017) with both adolescent and caregiver by a board-certified child psychiatrist, and validated self-report measures of depression symptoms, including the Brazilian Portuguese adaptation of the Mood and Feelings Questionnaire (MFQ) (Rosa et al., 2018). At the baseline assessment, we conducted structural and functional brain MRI scans and collected blood and saliva samples for measuring inflammatory proteins and performing RNAseq analyses, and isolated peripheral blood mononuclear cells (PBMCs) for future immune-related analyses (IDEA-FLAME study). Further information about the clinical measurements included in the baseline assessment and full description of the IDEA-RiSCo cohort have been published in a previous paper (Kieling et al., 2021).

Two online follow-ups with clinical questionnaires have also been conducted at 1- and 2-years following baseline assessment maintaining an exceptionally high retention rate (1-year: 95.33%, still ongoing 2-years: 96.09%). The 3-year face-to-face follow-up has been approved by the National Ethics Committee in Brazil and includes clinical assessment, smartphone-based passive and active sensing (nested study IDEA-TECH), brain MRI scans (nested study BRAIN-IDEA) and collection of blood samples (see Fig. 1). Local ethical approval for secondary data analyses is currently being sought in the UK and Italy where the laboratory analyses will take place and will be obtained before starting the laboratory work. The assessment of clinical symptoms at follow-up includes the administration of multiple scales (i.e., depressive symptoms measured with the Children's Depression Rating Scale-Revised (Poznanski et al., 1985), anxiety measured with the Spence Children's Anxiety Scale (Spence, 1998; DeSousa et al., 2014), irritability measured with the Affective Reactivity Index (Stringaris et al., 2012), and suicidality measured with the dimensional scales of the Columbia-Suicide Severity Rating Scale (Posner et al., 2011)). To assess rates of conversion to depression diagnosis, we will use the Brazilian Portuguese version of the K-SADS-PL (Caye et al., 2017). We will also assess whether participants sought any type of treatment for depression since baseline assessment.

### 3.3 Analyses to be conducted as part of the IDEA-FLAME study

For the nested IDEA-FLAME study we will conduct immune phenotyping of the IDEA-RiSCo cohort by 1) expanding the study of immune biomarkers at baseline, analysing immune cell markers in PBMCs previously collected and cryopreserved, and 2) repeating some of the measurement of inflammatory proteins and RNAseq analyses from blood samples collected at 3-year follow-up. Ethical approval has already been received in Brazil for collection of samples and analyses for IDEA-FLAME. Local ethical approval for secondary data analyses is currently being sought in the UK and Italy where the laboratory analyses will take place and will be obtained before starting the laboratory work.

#### 3.3.1 PBMCs separation and storage

At baseline assessment, blood was collected in two 4ml EDTA tubes by peripheral venipuncture, then carefully layered over conical Falcon tubes pre-filled with 1077-Histopaque for density gradient centrifugation according to the manufacturer's instructions. Tubes were centrifuged at 400×*g* for 30 minutes at room temperature with the brake off. Following aspiration of the top plasma layer, the buffy coat interphase layer containing the PBMCs was collected and washed three times in PBS at 250×*g* for 10 minutes at room temperature with brake on. Cell viability was evaluated by Trypan blue exclusion staining and found to exceed 90%. Cell pellet was then resuspended in 1 ml of cryoprotectant solution (90% fetal bovine serum and 10% DMSO) added drop wise over the cells. The cell suspension was transferred to a *Nunc* cryovial and stored in a Coolcell™ freezing container at −80 ​°C overnight. Finally, the samples were transferred into liquid nitrogen in the local laboratory in Brazil, before being shipped in liquid nitrogen to King's College London, where the flow cytometry analyses will take place.

#### 3.3.2 Multi-parameter flow cytometry analysis

To reveal immunopathological processes implicated in development of depression and establish phenotypic and functional differences between the three groups we will perform deep-immunophenotyping of PBMCs collected from participants at baseline. Using multi-parameter flow cytometry, we will investigate phenotypic and functional features of myeloid (monocytes, dendritic cells [DC]) and lymphoid (B cells, T cells, NK) immune cells, obtaining *i)* cell frequency of major populations and relative subsets (e.g., Tregs, Th1, Th2, Th17), *ii*) expression level of activation, maturation, adhesion and inhibitory markers, and *iii)* production of cytokines. We will run 3 high dimension 14-colours flow cytometry panels including: 1) a myeloid cell phenotypic panel (including surface and intracellular markers) to study activation (e.g., HLA-DR), maturation (CD80, CD83), adhesion (CD54) and inhibitory (PD-L1) markers; 2) a lymphoid phenotypic panel including a surface marker to study frequency and phenotype of B, T and NK cells by assessing activation (CD69); and 3) a lymphoid functional panel including phenotypic markers (as above) and cytokines (following stimulation for 5 hours by L phorbol-12-myristate-13-acetate and ionomycin in the presence of monensin and brefeldin) for the detection of intracellular cytokines (e.g. IFN-γ, TNF, IL-4,IL-17). Samples will be run on a BD FACS Fortessa x20 instrument at King's College London (KCL), where the PBMCs are currently stored, using Standardized Application Setup which allows combining of data acquired in different experiments, thus minimising batch-effects.

#### 3.3.3 Cytokines analyses

At the 3-year follow-up serum samples will be collected to measure a range of pro- and anti-inflammatory cytokines (e.g., IFN-γ, IL-1β, IL-2, IL-4, IL-6, IL-10, IL-17A and TNF) using high sensitivity multiplex arrays (i.e., V-plex pro-inflammatory panel 1 and cytokine panel 1 human kits) with a Meso Scale Discovery instrument. We will use the same technique and instrument used for measuring these cytokines in the baseline samples and already reported in detail in other publications (Enache et al., 2021; Nettis et al., 2021).

#### 3.3.4 RNA sequencing

At 3-year follow-up we will collect blood samples using PAXgene tubes for RNAseq analyses, and will follow the same protocol used for the baseline samples which resulted in good quality RNA samples. More specifically, RNA from all baseline samples have been isolated from PAXGene tubes following the manufacturer's instruction and were immediately stored at −80 ​°C for the subsequent analysis. The handling as well as the storage of the samples has been performed according to standard practice guidelines to avoid any deterioration. The quality of each sample has been checked by using Agilent Bioanalyzer 2100 and the RNA Integrity Number (RIN) has been checked for each sample. The RIN number is a software algorithm that indicates the RNA intactness by evaluation of the ribosomal ratio (18S and 28S) and it is based on a numbering system from 1 to 10, with 1 indicating a degraded profile and 10 the most intact (Mueller and Schröder, 2004); for genome-wide gene expression analysis, the RIN value is considered to be good when it falls within the range of 7–10. At baseline, 146 out of 150 samples (93% of the entire cohort of samples) had a RIN value above 7.5, indicating intact and very good quality RNA; moreover, among these, 97 samples show RIN >9.

RNA sequencing analyses will be run at the University of Milan, Italy, where the first baseline samples have been analysed in order to use the same instrument. Briefly, RNA-Sequencing libraries will be prepared using the TrueSeq Stranded Total RNA Sample Preparation kit (Illumina), using the Ribo-Zero Gold kit (Illumina) for depletion of ribosomal RNA, followed by first and second strand cDNA synthesis and fragmentation of dsDNA. Then, fragmented DNA will be used for A-tailing, adaptor ligation and 12 cycles of PCR amplification. Libraries will be quantified using high sensitivity chip on a Labchip (PerkinElmer), quantitative PCR (KAPA Library Quantification, Kapa Biosystems), and PicoGreen (Life Technologies). Three libraries will be run per lane of an Illumina HiSeq 2000 (100bp paired-end), which should yield ≈60 million reads/library.

### 3.4 Statistical analyses

For aim 1, we will use generalized linear models to examine whether risk group classification (high-risk, low-risk, depressed) is associated with differences in cellular immune markers at baseline. To reduce the likelihood of overfitting to our sample and increase the possibility of generalising our findings to other datasets, we will conduct stratified 5-fold cross-validation in all of our statistical analyses (with ∼10 participants from each of three risk groups randomly assigned to each of the 5 subsamples for testing the models). For aim 2, we will test associations between baseline cytokine levels and clinical presentation at 3-year follow-up, while controlling for baseline depression symptoms, by using partial correlation. We will also control for whether participants sought any treatment in the intervening period, and examine whether this moderates the association between risk group status at baseline and follow-up depression symptoms. For both aim 1 and 2, we will test the contribution of potential confounders (smoking, obesity, medical comorbidities) by including each variable in turn in the model (general linear model for aim 1 and partial correlation for aim 2) and see whether any identified association would remain statistically significant after introducing each confounder. To explore the potential role of other factors influencing the immune phenotype, we will also conduct explorative correlation analyses to test associations of factors such as relationship/attachment with parents with the immune markers identified to be specifically relevant for the onset of depression. For the bioinformatic analyses for RNAseq data: as for the baseline, we will use FastQC for checking the quality of the data; FASTX-Toolkit and Trimmomatic for adapter and over-represented sequences trimming; Bowtie2 for alignment; TopHat for transcript counting; and DESeq2 for differential expression analysis. Following high-throughput sequencing, 100bp paired-end reads will be aligned to the hg19 human genome using TopHat v2.1.0 (http://tophat.cbcb.umd.edu/) with a mate insert distance of 75 bp (-r) and library type fr-unstranded. Reads passing a mapping quality of at least 50 will be used for gene and transcript quantification and DESeq2 will be used for differential expression analysis. Data will also be imported in Ingenuity Pathway Analyses Software (IPA) and Gene Mania Software in order to identify pathways which are differentially modulated.

### 3.5 Power analysis

Prior research has shown differences in lymphocyte profiles (CD4^+^ & CD38^+^ T cells) between healthy adolescents and adolescents with experience of traumatic events with an effect size of Cohen's d ​= ​0.88 (do Prado et al., 2017). Another previous flow cytometry study in adult patients with depression vs healthy controls showed a difference in monocyte counts with a higher number of monocytes in patients with depression vs health controls with an effect size of d ​= ​0.65 (Euteneuer et al., 2017). The baseline sample includes 150 risk-stratified adolescents (50 per group). Considering 50 participants per group, using G∗Power we computed a power of 89.6% to detect differences in immune cell populations with a similar effect size (d ​= ​0.65, using a two-tailed p value of 0.05) between the depressed group and low-risk group and between the high-risk group and low-risk group for the analyses for our aim 1.

For the longitudinal analyses, based on retention rates of ∼85% for the 1993 Pelotas study, we assume a retention rate of around 80% at three-year follow-up, this will result in a total sample of 120 adolescents (approximately 40 participants per group). In our previous work (Lynall et al., 2019), we found a correlation between a Principal Component score (mostly weighted on myeloid cells and CD4^+^ T cells) and severity of depressive symptoms with a ρ ​= ​0.26. For aim 2, considering a sample of 120 subjects, using G∗Power we computed a power of 83% to detect a similar effect size (using a two-tailed p value of 0.05) for a correlation between immune markers and severity of depressive symptoms. For aim 3, focusing on RNAseq analyses and by using the ssizeRNA package we determined that a sample size of 40 per group has a power of 92% for detecting significantly different expressed genes, with FC of 2, FDR ​= ​0.05 and considering 15000 as the number of detected genes and 0.1 as the dispersion parameter for each gene (Bi and Liu, 2016).

## 4 Discussion

The IDEA-FLAME study is the first project aiming to perform deep immune phenotyping and whole-genome transcriptomic analysis of adolescents with or at risk of depression. It has the advantage of leveraging on a unique risk-stratified cohort of adolescents and addressing the issues of comparing adolescents with depression with a potentially widely heterogenous sample of controls. Understanding immune cells and pathways involved in the development and increased risk of adolescent depression will help to better identify adolescents at high risk of developing depression at an early stage and potential key therapeutic targets to inform the development of interventions to prevent onset of depression among adolescents worldwide. These steps are extremely important to improve our understanding of adolescent depression and contribute to the development of effective early interventions that would potentially reduce the burden of depression in adolescents in the future.

The results from this study will support future work that in the long-term could lead to: 1) prediction of clinical outcomes/trajectory, 2) identification of immune targets for developing specific anti-inflammatory interventions suitable for adolescent depression, and 3) identification of distinct subtypes of adolescents who would potentially benefit from anti-inflammatory treatment strategies. The development of personalised early intervention strategies guided by immune mechanistic biomarkers will improve identification of adolescents at risk at an early stage, support targeted prevention efforts, and better treatment for adolescent depression. Ultimately, a better understanding of the immune mechanisms underlying adolescent depression and the identification and characterization of adolescents at higher risk of developing depression could potentially contribute to future prevention and treatment strategies leading to a reduction in the incidence of depression among adolescents and adults across the globe.

## Funding

The IDEA project is funded by an MQ Brighter Futures grant [MQBF/1 IDEA] with additional funding from MQ for the IDEA-FLAME study. Additional support was provided by the UK Medical Research Council [MC_PC_MR/R019460/1] and the Academy of Medical Sciences [GCRFNG∖100281] under the Global Challenges Research Fund. The BRAIN-IDEA study is supported by the U.S. National Institute of Mental Health [R21MH124072-01]. The IDEA-TECH study is supported by the UK Royal Academy of Engineering under the Frontiers Follow-on Funding scheme [FF∖1920∖1∖61]. Dr Valeria Mondelli is supported by the National Institute for Health Research (NIHR) Biomedical Research Centre at South London and Maudsley NHS Foundation Trust and King's College London. Research in Dr Paola Di Meglio ‘s laboratory is supported by the National Institute for Health Research (NIHR) Biomedical Research Centre based at Guy's and St Thomas' NHS Foundation Trust and King's College London. Dr Kieling is a Conselho Nacional de Desenvolvimento Científico e Tecnológico (CNPq) researcher and an Academy of Medical Sciences Newton Advanced Fellow. Dr. Fisher is part supported by the Economic and Social Research Council (ESRC) Centre for Society and Mental Health at King's College London [ES/S012567/1].

## Role of funding sources

These funders played no role in study design; in the collection, analysis and interpretation of data; in the writing of the report; nor in the decision to submit this article for publication. The views expressed are those of the authors and not necessarily those of the funders, the NHS, the NIHR, the Department of Health and Social Care, the ESRC, or King's College London.

## Declaration of competing interest

Dr Mondelli has received research funding from Johnson & Johnson as part of a research program on depression and inflammation, but the research described in this paper is unrelated to this funding. All other authors declare they have no conflicts of interest to report.
